# Interleukin 35 Polymorphisms Are Associated with Decreased Risk of Premature Coronary Artery Disease, Metabolic Parameters, and IL-35 Levels: The Genetics of Atherosclerotic Disease (GEA) Study

**DOI:** 10.1155/2017/6012795

**Published:** 2017-02-22

**Authors:** Rosalinda Posadas-Sánchez, Nonanzit Pérez-Hernández, Javier Angeles-Martínez, Fabiola López-Bautista, Teresa Villarreal-Molina, José Manuel Rodríguez-Pérez, José Manuel Fragoso, Carlos Posadas-Romero, Gilberto Vargas-Alarcón

**Affiliations:** ^1^Department of Endocrinology, Instituto Nacional de Cardiología Ignacio Chávez, Mexico City, Mexico; ^2^Department of Molecular Biology, Instituto Nacional de Cardiología Ignacio Chávez, Mexico City, Mexico; ^3^Cardiovascular Genomics Laboratory, Instituto Nacional de Medicina Genómica (INMEGEN), Mexico City, Mexico

## Abstract

Interleukin 35 (IL-35) is a heterodimeric cytokine involved in the development of atherosclerosis. The aim of the present study was to establish if the polymorphisms of* IL-12A* and* EBI3* genes that encode the IL-35 subunits are associated with the development of premature coronary artery disease (CAD) in Mexican individuals. The* IL-12A* and* EBI3* polymorphisms were determined in 1162 patients with premature CAD and 873 controls. Under different models, the* EBI3* rs428253 (OR = 0.831, *P*_add_ = 0.036; OR = 0.614, *P*_rec_ = 0.033; OR = 0.591, *P*_cod2_ = 0.027) and* IL-12A* rs2243115 (OR = 0.674, *P*_add_ = 0.010; OR = 0.676, *P*_dom_ = 0.014; OR = 0.698, *P*_het_ = 0.027; OR = 0.694, *P*_cod1_ = 0.024) polymorphisms were associated with decreased risk of developing premature CAD. Some polymorphisms were associated with clinical and metabolic parameters. Significant different levels of IL-35 were observed in* EBI3* rs4740 and rs4905 genotypes only in the group of healthy controls. In summary, our study suggests that the* EBI3* and* IL-12A* polymorphisms play an important role in decreasing the risk of developing premature CAD; it also demonstrates the relationship of the* EBI3* rs4740 and rs4905 genotypes with IL-35 levels in healthy individuals.

## 1. Introduction

Atherosclerosis is a progressive and multifactorial disease influenced by genetic and environmental factors. A major consequence of the atherosclerosis is the coronary artery disease (CAD). It is well known that inflammation plays an important role in the pathogenesis of atherosclerosis and its complications [1]. The inflammatory phenomenon begins when circulating low density lipoprotein (LDL) particles present in the subendothelial space are oxidized, acquiring proinflammatory properties [2]. Depositions of circulating monocytes/macrophages exacerbate the inflammatory response, because the arterial proteoglycans retain and modify the lipoproteins, increasing their phagocytosis into macrophages. In addition, cell recruitment, production of adhesion molecules, chemokines, and cytokines all cause increased atheroma volume [3]. Aside from the classic cytokines known to be involved in the inflammatory process, a new cytokine, interleukin 35, has recently been described, which also plays a significant role in this phenomenon [4]. Interleukin- (IL-) 35 is a heterodimeric cytokine composed of the Epstein-Barr virus-induced 3 (EBI3) and p35 subunits; it belongs to the IL-6/IL-12 cytokine family that includes IL-12, IL-23, IL-27, and IL-35 molecules [5]. Unlike TGF*β*, but similar to IL-10 and IL-27, IL-35 is minimally expressed in human tissues and is mainly induced in inflammatory conditions [4]. Unlike the other members of the IL-12 family, IL-35 is predominantly secreted by regulatory T cells (Treg). As a matter of fact, it has been shown that this cytokine represses T-cell proliferation and function in several in vitro and in vivo disease models [6–8]. Some studies have reported that IL-35 inhibits several inflammatory disorders, such as inflammatory bowel disease [9], autoimmune encephalomyelitis [10], autoimmune diabetes [11], and collagen II-induced arthritis [12]. On the other hand, decreased levels of IL-35 have been reported in patients with acute coronary syndrome (unstable angina pectoris and acute myocardial infarction) compared with a chest pain syndrome group [13]. This finding and the fact that IL-35 is strongly expressed in atherosclerotic plaque [14] suggest that this cytokine could be involved in the development of atherosclerosis. In an animal model, Wang et al. [15] have recently demonstrated the role of IL-35 in the development of atherosclerosis. Apolipoprotein E-deficient (apoE^−/−^) mice with an established atherosclerotic lesion displayed a lower level of IL-35 compared to the age-matched wild type C57BL/6 mice without plaque. On the other hand, the expression of the IL-35 increased significantly in apoE^−/−^ mice with attenuated plaque.

The* IL-12A* gene encodes the p35 subunit of IL-35; it is located on chromosome 3q25.33 and consists of seven exons. Several polymorphisms have been described in the* IL-12A* gene and some of them have been associated with susceptibility to Graves' and Alzheimer's disease [16, 17]. The *β* subunit (EBI3) of IL-35 is encoded by* EBI3* gene located on chromosome 19q13.3 and contains 5 exons. Zhang et al. reported that the* EBI3* rs428253 polymorphism was associated with decreased risk of development of chronic rhinosinusitis and allergic rhinitis [18, 19]. Currently, no studies have examined the role of the polymorphisms present in the* IL-12A* and* EBI3* genes regarding the susceptibility or protection to the development of CAD. Thus, the aim of the present study was to establish the effect of these polymorphisms in the genetic susceptibility to development of premature CAD in Mexican individuals. Based on the results obtained with a functional prediction analysis, we decided to study four polymorphisms from the* IL-12A *gene (rs2243115, rs2243123, rs583911, and rs568408) and three from the* EBI3 *gene (rs428253, rs4740, and rs4905) with possible functional consequences and/or with minor allele frequency > 5%. The* IL-12A *rs2243115 polymorphism produces binding sites for the transcription factors AP2, LRH1, and SF1, whereas the* IL-12A *rs568408 polymorphism produces binding sites for microRNAs. Further, the* EBI3* rs428253 produces a binding site for LEF1 factor and rs4740 for SR proteins. In spite of the fact that the rs4905 (*EBI3* gene), rs2243123, and rs583911 (*IL-12A* gene) polymorphisms were not functional, they were informative (minor allele frequency > 5%) and were therefore included in the study.

## 2. Materials and Methods

### 2.1. Subjects

The study complies with the Declaration of Helsinki and was approved by the Ethics Committee of the Instituto Nacional de Cardiología Ignacio Chávez (INCICH). All participants provided written informed consent. The study included 1162 patients with premature CAD and 873 healthy controls belonging to the Genetics of Atherosclerotic Disease (GEA) Mexican Study. Premature CAD was defined as history of myocardial infarction, angioplasty, revascularization surgery, or coronary stenosis > 50% on angiography, diagnosed before age of 55 in men and before age of 65 in women. Controls were apparently healthy asymptomatic individuals without family history of premature CAD, recruited from blood bank donors and through brochures posted in Social Service centers. Chest and abdomen computed tomographies were performed using a 64-channel multidetector helical computed tomography system (Somatom Sensation, Siemens) and interpreted by experienced radiologists. Scans were read to assess and quantify the following: (1) coronary artery calcification (CAC) score using the Agatston method [20] and (2) total adipose tissue (TAT) and subcutaneous and visceral adipose tissue areas (SAT and VAT) as described by Kvist et al. [21]. For the present study, the control group only included individuals with CAC = 0, who were nondiabetic, and with normal glucose levels (*n* = 873). In the whole sample, the demographic, clinical, anthropometric, and biochemical parameters and cardiovascular risk factors were evaluated and defined as previously described [22–24]. Briefly, hypercholesterolemia was defined as total cholesterol (TC) levels ≥ 200 mg/dL. Hypertension was defined as systolic blood pressure ≥ 140 mmHg and/or diastolic blood pressure ≥ 90 mmHg or the use of oral antihypertensive therapy. Type 2 diabetes mellitus (T2DM) was defined with a fasting glucose ≥ 126 mg/dL and was also considered when participants reported glucose-lowering treatment or a physician diagnosis of T2DM. Obesity was defined as body mass index (BMI) ≥ 30 kg/m^2^. Hypoalphalipoproteinemia, hypertriglyceridemia, and metabolic syndrome (MS) were defined using the criteria from the American Heart Association, National Heart, Lung, and Blood Institute Scientific Statement [25], except for central obesity that was considered when waist circumference was 90 cm in men and 80 cm in women [26]. Hyperuricemia was considered with a serum uric acid > 6.0 mg/dL and >7.0 mg/dL for women and men, respectively [27]. Insulin resistance was estimated using the homeostasis model assessment of insulin resistance (HOMA-IR). The presence of insulin resistance was considered when the HOMA-IR values were ≥75th percentile (3.66 in women and 3.38 in men). Hyperinsulinemia was defined when insulin concentration was ≥75th percentile (16.97 *μ*IU/mL in women and 15.20 *μ*IU/mL in men). Hypoadiponectinemia was defined when adiponectin concentration was ≤25th percentile (8.67 *μ*g/mL in women and 5.30 *μ*g/mL in men). Increased VAT was defined as VAT ≥ 75th percentile (122.0 cm^2^ in women and 151.5 cm^2^ in men) and increased SAT as SAT ≥ 75th percentile (335.5 cm^2^ in women and 221.7 cm^2^ in men). Elevated alanine aminotransferase (ALT) was defined as ALT activity ≥ 75th percentile (21.0 IU/L in women and 24.5 IU/L in men). Elevated aspartate aminotransferase (AST) was defined as AST activity ≥ 75th percentile (25 IU/L in women and 28 IU/L in men) and elevated gamma glutamyltransferase (GGT) was defined as GGT ≥ 75th percentile (21.0 IU/L in women and 27.5 IU/L in men). These cutoff points were obtained from a GEA study sample of 131 men and 185 women without obesity and with normal values of blood pressure, fasting glucose, and lipids.

All GEA participants are unrelated and of self-reported Mexican-Mestizo ancestry (three generations). In order to establish the ethnical characteristics of the studied groups, we analyzed 265 ancestry informative markers (AIMs). Using the ADMIXTURE software, the Caucasian, Amerindian, and African backgrounds were determined. Similar background in premature CAD patients and healthy controls was found (*P* > 0.05). Patients showed 55.8% of Amerindian ancestry, 34.3% of Caucasian ancestry, and 9.8% of African ancestry, whereas controls showed 54.0% of Amerindian ancestry, 35.8% of Caucasian ancestry, and 10.1% of African ancestry.

### 2.2. IL-35 Levels Determination

Considering that obesity is frequently associated with a chronic low grade inflammatory process, which could modify the cytokine levels, plasma concentration of IL-35 was determined in a subsample of nonobese subjects with normal values (<3 mg/L) of high sensitivity C reactive protein (hsCRP) (451 premature CAD patients and 458 healthy controls) using a Bioplex system (Bio-Rad, Contra Costa County, State of California, USA) according to manufacturer's instructions.

### 2.3. Genetic Analysis

The 5′ exonuclease TaqMan genotyping assays were used to determine the* IL-12A* (rs2243115, rs568408, rs2243123, and rs583911) and* EBI3* (rs428253, rs4740, and rs4905) polymorphisms. The determinations were made on an ABI Prism 7900HT Fast Real-Time PCR system, according to manufacturer's instructions (Applied Biosystems, Foster City, CA, USA). Samples previously sequenced of the different genotypes of the polymorphisms studied were included as positive controls.

### 2.4. Functional Prediction Analysis

In order to predict the potential effect of the* IL-12A *and* EBI3 *polymorphisms, we used the following bioinformatics tools: FastSNP [28], SNP Function Prediction (http://snpinfo.niehs.nih.gov/snpinfo/snpfunc.html), Human-transcriptome Database for Alternative Splicing (http://www.h-invitational.jp/h-dbas/), Splice Port: An Interactive Splice Site Analysis Tool (http://spliceport.cbcb.umd.edu/SplicingAnalyser.html), ESE finder (http://rulai.cshl.edu/cgi-bin/tools/ESE3/esefinder.cgi), HSF (http://www.umd.be/HSF/), and SNPs3D (http://www.snps3d.org/).

### 2.5. Statistical Analysis

The analysis was made using the SPSS version 15.0 statistical package (SPSS, Chicago, Il). Means, medians, interquartile ranges, and frequencies were calculated as the case may be. Continuous and categorical variables were analyzed by *t*-Student's test, Mann–Whitney *U* test, Kruskal-Wallis, and Chi square or Fisher test as appropriate. The polymorphism associations with premature CAD and other variables were analyzed using logistic regression under the following inheritance models: additive (major allele homozygotes versus heterozygotes versus minor allele homozygotes), codominant 1 (major allele homozygotes versus heterozygotes), codominant 2 (major allele homozygotes versus minor allele homozygotes), dominant (major allele homozygotes versus heterozygotes + minor allele homozygotes), heterozygous (heterozygotes versus major allele homozygotes + minor allele homozygotes), and recessive (major allele homozygotes + heterozygotes versus minor allele homozygotes). For the* EBI3* polymorphisms all the inheritance models were adjusted for age, gender, BMI, current smoking status, ALT, AST, and uric acid. For the* IL12A* polymorphisms, models were adjusted for age, gender, BMI, and current smoking status. Genotype frequencies did not deviate from Hardy-Weinberg equilibrium in any case (HWE, *P* > 0.05).

## 3. Results

Tables 1 and 2 exhibit the clinical and demographic characteristics of the studied individuals. As we can see, a number of differences were observed between premature CAD patients and healthy controls. As shown in Table 1, the systolic and diastolic blood pressure are both low and within normal limits; however, some of our patients have hypertension (Table 2). The reason for this discrepancy is that some patients with hypertension are under treatment and in consequence their pressure levels were within normal range. As expected, hypercholesterolemia [TC > 200 mg/dL or low density lipoprotein cholesterol (LDL-C) ≥ 130 mg/dL], inflammation [defined as hsCRP levels ≥ 3 mg/L], and current smoking habit were significantly more frequent in controls than in premature CAD patients most likely due to the effect of statin treatment and a life style changes advice after the cardiovascular event.

### 3.1. Association of the* EBI3 *and* IL-12A* Polymorphisms with Premature CAD

The distribution of the* EBI3* (rs4740 and rs4905) and* IL-12A* (rs2243123, rs568408, and rs583911) polymorphisms was similar in premature CAD and healthy controls. However, under additive, recessive, and codominant 2 models, the* EBI3* rs428253 polymorphism was associated with decreased risk of developing premature CAD (*P*_add_ = 0.036, *P*_rec_ = 0.033, and *P*_cod2_ = 0.027). The models were adjusted for age, gender, BMI, current smoking status, ALT, AST, and uric acid. In the same way, the* IL-12A* rs2243115 (*P*_add_ = 0.010, *P*_dom_ = 0.014, *P*_het_ = 0.027, and *P*_cod1_ = 0.024) polymorphism was associated with diminished risk of developing premature CAD (Table 3) under different models adjusted for age, gender, BMI, and current smoking status.

### 3.2. Association of the* EBI3* and* IL-12A* Polymorphisms with Metabolic Parameters

In premature CAD patients under different models, the* EBI3* rs428253 polymorphism was associated with high levels of ALT > p75 (*P*_add_ = 0.006, *P*_dom_ = 0.004, *P*_het_ = 0.010, and *P*_cod1_ = 0.006) and AST > p75 (*P*_cod2_ = 0.042) and with decreased risk of developing T2DM (*P*_dom_ = 0.033, *P*_het_ = 0.022, and *P*_cod1_ = 0.022). The* EBI3* rs4905 polymorphism was associated with high levels of ALT > p75 (*P*_add_ = 0.023, *P*_dom_ = 0.024, and *P*_cod1_ = 0.045). Additionally, the* IL-12A* rs2243123 polymorphism was associated with increased risk of T2DM (*P*_rec_ = 0.021, *P*_cod2_ = 0.028), while the rs2243115 polymorphism correlated with reduced risk of metabolic syndrome (*P*_add_ = 0.015, *P*_dom_ = 0.017, *P*_het_ = 0.022, and *P*_cod1_ = 0.021). The rs583911 polymorphism was linked with diminished levels of inflammation (hsCRP ≥ 3 mg/L, *P*_rec_ = 0.017), high levels of AST > p75 (*P*_add_ = 0.013, *P*_dom_ = 0.046, *P*_rec_ = 0.035, and *P*_cod2_ = 0.013), and high levels of GGT > p75 (*P*_rec_ = 0.042) (Table 4).

In healthy controls, the* EBI3* rs428253 polymorphism was associated with the presence of hyperuricemia (*P*_het_ = 0.024, *P*_cod1_ = 0.032), the* EBI3* rs4740 was associated with decreased risk of central obesity (*P*_het_ = 0.035, *P*_cod2_ = 0.038) and with increased risk of high levels of AST > p75 (*P*_add_ = 0.046, *P*_dom_ = 0.014, *P*_het_ = 0.015, and *P*_cod1_ = 0.011), and the* EBI3* rs4905 was linked with reduced risk of central obesity (*P*_rec_ = 0.040, *P*_cod2_ = 0.046) and increased risk of high levels of AST > p75 (*P*_dom_ = 0.020, *P*_het_ = 0.020, and *P*_cod1_ = 0.016). In addition, we found that the* IL-12A* rs568408 correlated with decreased risk of metabolic syndrome (*P*_add_ = 0.042) and the* IL-12A* rs583911 was associated with high levels of SAT (*P*_het_ = 0.004, *P*_cod1_ = 0.017) (Table 5).

### 3.3. Association of the* EBI3* and* IL-12A* Genotypes with IL-35 Levels

**Figure 1:**
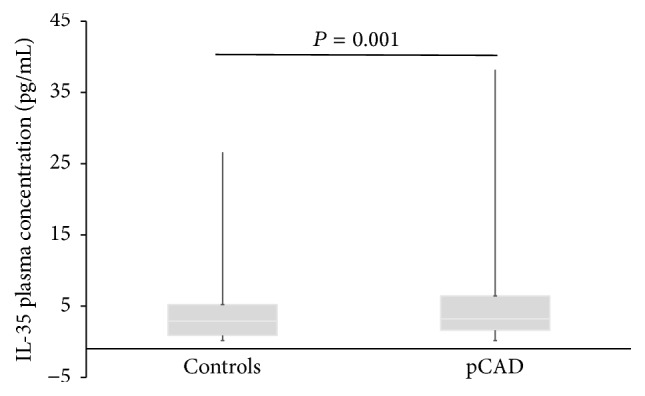
Interleukin 35 plasma concentration in 477 control subjects and 447 premature CAD (pCAD) patients. Comparisons were made using Mann–Whitney *U* test. Premature CAD patients have significantly higher IL-35 levels than control subjects (3.2 [1.6–6.7] pg/mL versus 2.7 [0.8–5.2] pg/mL, respectively, *P* = 0.001).

The levels of IL-35 were determined in 451 premature CAD patients and in 458 healthy controls. Individuals with extreme outliers values were not included in the analysis (4 patients and 11 controls).  Figure 1 shows that premature CAD patients have significantly higher IL-35 levels than control subjects (3.2 [1.6–6.7] pg/mL versus 2.7 [0.8–5.2] pg/mL, respectively, *P* = 0.001, Figure 1). Additionally, we found that, in the healthy control group, significant different levels of IL-35 were observed in* EBI3* rs4740 (*AA* = 3.40 [0.88–7.90] pg/mL,* GA* = 3.00 [1.63–5.23] pg/mL, and* GG* = 2.52 [0.88–4.50] pg/mL; *P* = 0.020) and rs4905 (*GG* = 3.78 [0.88–7.90] pg/mL,* AG* = 3.00 [1.63–5.23] pg/mL, and* AA* = 2.52 [0.88–4.40] pg/mL; *P* = 0.017) genotypes (Table 6).

## 4. Discussion

Interleukin-35 is a heterodimeric cytokine that belongs to the IL-6/IL-12 family and is composed of two chains (p35 and EBI3): one encoded by the* IL-12A* (p35) gene and the other by the* EBI3* gene. This cytokine has been associated with the development of several inflammatory diseases. In fact, a recent study on this molecule points out its probable protective role against atherosclerosis [15]. The role of the IL-35 in the inflammatory diseases suggests that the genes that encode its different subunits could be candidates in the study of atherosclerosis and its complications (e.g., CAD). To the best of our knowledge, this is the first study that evaluates the role of* IL-12A* and* EBI3* polymorphisms in premature CAD. In this report, we found that two polymorphisms, namely,* EBI3* rs428253 and* IL-12A* rs2243115, were associated with reduced risk of developing premature CAD. These polymorphisms were also associated with decreased risk of T2DM (*EBI3* rs428253) and metabolic syndrome (*IL-12A *rs2243115) in premature CAD patients. However, in healthy controls only the* EBI3* rs428253 correlates with increased risk of hyperuricemia. The polymorphisms that were not linked with risk of premature CAD were associated with other clinical and metabolic parameters. In premature CAD patients, the* EBI3* rs4905 was related to high levels of ALT, the* IL-12A* rs2243123 was associated with increased risk of T2DM, and* IL-12A* rs583911 correlated with inflammation, high levels of AST, and GGT. In healthy controls, the* EBI3 *rs4905 and* EBI3 *rs4740 were associated with low risk of central obesity and increased risk of high levels of AST, whereas the* IL-12A *rs583911 correlated with high risk of increased SAT and* IL-12A *rs568408 with diminished risk of metabolic syndrome. According to the informatics tools, the two polymorphisms, which were associated with decreased risk of developing premature CAD, have a possible functional effect. Specifically, the* EBI3* rs428253 modifies a binding site for the lymphoid enhancer-binding factor 1 (LEF1) that is a decisive transcription factor in the control of the granulopoiesis proliferation, proper lineage commitment, and granulocytic differentiation [29]. Furthermore, the* IL-12A* rs2243115 polymorphism, located in the promoter region, produces binding sites for the transcription factors AP2, LRH1, and SF1. Thus, after considering that the studied polymorphisms could have an effect in the production of IL-35, we analyzed the molecule serum levels in a group of premature CAD patients and healthy controls. Coronary patients showed significantly higher IL-35 levels than control subjects; however, the difference was small. We cannot define whether these differences could have an effect on the development of atherosclerosis. As we know, atherosclerosis is a multifactorial disease and multiple cytokines, both pro- and anti-inflammatory, play a role in the genesis and progression of the inflammatory process. In this analysis, neither* EBI3 *rs428253 nor* IL-12A* rs2243115 (the polymorphisms associated with premature CAD with a possible functional effect) showed a correlation with IL-35 serum levels. The fact that the associated polymorphisms with decreased risk of developing premature CAD did not correlate with IL-35 levels could be explained considering that the production of IL-35 and other molecules is a complex mechanism that involves not only changes at DNA level but also epigenetics modifications. Moreover, it is important to considered that in our study the levels of IL-35 were measured in circulation and not at the lesion site. On the other hand, the* EBI3* rs4740 and rs4905 polymorphisms were associated with different levels of IL-35. Furthermore, this association was observed only in the healthy control groups. From these two polymorphisms, only* EBI3* rs4740 was functional according to the informatics tools. Interestingly, this polymorphism produces binding sites for Srp40, and SRp55, which belong to the family of SR proteins that regulate alternative splicing [30].

IL-35 is a heterodimeric cytokine that belongs to the IL-6/IL-12 cytokine family, which includes IL-12, IL-23, IL-27, and IL-35 molecules. These cytokines share subunits that are encoded by* EBI3*,* IL-12A*,* IL-12B*,* IL-23A*, and* IL27p28* genes. Our research group is studying several polymorphisms located in these genes in order to establish its role in the genetic susceptibility to developing premature CAD and cardiovascular risk factors. At the moment, we have analyzed the polymorphisms of the* IL27p28* gene that encode the p28 subunit of the IL-27. This analysis showed that two polymorphisms of this gene (rs26528 and rs40837) were significantly associated with a lower risk of premature CAD. Using the luciferase assay we demonstrate that the rs40837 polymorphism has a functional effect. In this study, we also determined independently the levels of IL-27. None of the studied polymorphisms were associated with IL-27 levels (personal communication).

*IL-12A* polymorphisms have been associated with the development of several diseases, such as rheumatoid arthritis [31], Alzheimer's disease [17], Graves' disease [16], and asthma [32]. In contrast,* EBI3* polymorphisms have been associated with ulcerative colitis [33], pulmonary tuberculosis [34], chronic rhinosinusitis [19], and allergic rhinitis [18]. In these studies,* IL-12A* and* EBI3* genes were analyzed independently. To the best of our knowledge, no studies so far have reported an analysis, in which both genes have been analyzed in concert for any disease.

As for the limitations, herein, we have only included the study of four polymorphisms of* IL-12A *and three of the* EBI3* gene, which seem to be functional and/or informative based on the analysis of the prediction software results. Since this is the first work that documents the correlation of the* IL-35* polymorphisms with premature CAD, and cardiovascular parameters, further studies in an independent group of patients are mandatory to validate the results. It is important to note that one strength of our work is that the control group only included individuals without subclinical atherosclerosis (i.e., individuals without coronary artery calcification).

## 5. Conclusion

In summary, our results indicate that there exists a statistically significant association between the* EBI3* rs428253 and* IL-12A* rs2243115 polymorphisms and a reduced risk of developing premature CAD. Some of the studied polymorphisms were associated with cardiovascular parameters. The* EBI3* rs4740 and* EBI3 *rs4905 genotypes were associated with a variation in IL-35 serum levels in healthy controls. To the best of our knowledge, this is the first study that evaluates the role of* IL-12A* and* EBI3* polymorphisms in premature CAD. For this reason, the detected associations are not yet definitive, and replicate studies in independent populations are warranted to confirm these findings.

## Figures and Tables

**Table 1:** Clinical and metabolic characteristics of the studied groups.

	Control (*n* = 873)	Premature CAD (*n* = 1162)	*P*
Age (years)	51 ± 9	54 ± 8	<0.001
Gender (% male)	40.7	81.1	<0.001
Body mass index (kg/m^2^)	27.3 [24.9–30.2]	28.3 [25.9–31.1]	<0.001
Waist circumferences (cm)	92 ± 11	98 ± 10	<0.001
Systolic blood pressure (mmHg)	111 [103–121]	116 [106–127]	<0.001
Diastolic blood pressure (mmHg)	70 [65–76]	71 [66–78]	0.001
Total adipose tissue (cm^2^)	416 [330–514]	425 [340–523]	0.147
Visceral adipose tissue (cm^2^)	130 [98–172]	168 [129–215]	<0.001
Subcutaneous adipose tissue (cm^2^)	280 [209–356]	245 [193–313]	<0.001
Total cholesterol (mg/dL)	190 [167–210]	160 [132–193]	<0.001
High density lipoprotein cholesterol (mg/dL)	46 [37–56]	37 [32–44]	<0.001
Low density lipoprotein cholesterol (mg/dL)	116 [95–133]	91 [68–116]	<0.001
Triglycerides (mg/dL)	138 [102–190]	162 [119–219]	<0.001
Non-HDL-cholesterol (mg/dL)	141 [119–162]	120 [93–151]	<0.001
Alanine aminotransferase (IU/L)	23 [17–32]	26 [19–36]	<0.001
Aspartate aminotransferase (IU/L)	24 [20–30]	26 [22–31]	<0.001
Glucose (mg/dL)	87 [82–92]	95 [87–117]	<0.001
Insulin (*µ*IU/mL)	16 [12–21]	20 [15–28]	<0.001
Homeostasis model assessment of insulin resistance	3.3 [2.4–4.7]	5.1 [3.5–7.7]	<0.001
High sensitivity C reactive protein (mg/L)	1.4 [0.7–2.9]	1.2 [0.6–2.6]	0.005
Adiponectin (*µ*g/mL)	8.5 [5.3–13.6]	5.2 [3.2–8,1]	<0.001
Uric acid (mg/dL)	5.3 [4.3–6.3]	6.5 [5.4–7.4]	<0.001

Data are shown as mean ± standard deviation, median [interquartile range], or percentage. Comparisons were made using Student's *t*-test or Mann–Whitney *U* test, as appropriate, for continuous variables, and by Chi square analysis for categorical variables. CAD: coronary artery disease.

**Table 2:** Cardiovascular risk factors prevalence in the study population.

	Control (*n* = 873)	Premature CAD (*n* = 1162)	^*∗*^ *P*
Total cholesterol > 200 mg/dL (%)	36.3	20.3	<0.001
LDL-cholesterol ≥ 130 mg/dL (%)	29.2	16.1	<0.001
Hypoalphalipoproteinemia (%)	49.3	67.2	<0.001
Hypertriglyceridemia (%)	42.8	56.2	<0.001
Non-HDL-cholesterol > 160 mg/dL (%)	26.0	19.5	<0.001
Obesity (%)	26.1	35.0	<0.001
Abdominal obesity (%)	77.6	83.6	<0.001
Type 2 diabetes mellitus (%)	0	35.4	<0.001
Hyperinsulinemia (%)	45.8	71.4	<0.001
Insulin resistance (%)	44.2	77.0	<0.001
Metabolic syndrome (%)	29.7	71.9	<0.001
Hypertension (%)	5.7	68.1	<0.001
High visceral adipose tissue (%)	49.8	64.6	<0.001
Current smoking status (%)	23.5	11.6	<0.001
Hypoadiponectinemia (%)	40.0	56.5	<0.001
High sensitivity C reactive protein ≥ 3 mg/L (%)	23.6	21.3	0.114
Hyperuricemia (%)	16.8	35.9	<0.001

Data is shown as percentage. ^*∗*^Comparisons were made using Chi square analysis. CAD: coronary artery disease, LDL: low density lipoprotein, and HDL: high density lipoprotein.

**Table 3:** Association between *EBI3* and *IL-12A* gene polymorphisms and premature coronary artery disease.

Polymorphism	Genotype frequency *n* (%)			MAF	Model	OR [95% CI]	*P*
(i) *EBI3*^**∗**^							
*rs428253*	*G>C*						
	*GG*	*GC*	*CC*				
Control (*n* = 873)	536 (0.614)	277 (0.317)	60 (0.069)	0.227	*Additive*	*0.831 [0.699–0.988]*	*0.036*
					Dominant	0.842 [0.681–1.042]	0.115
					*Recessive*	*0.614 [0.392–0.963]*	*0.033*
pCAD (*n* = 1162)	740 (0.637)	371 (0.319)	51 (0.044)	0.204	Heterozygote	0.935 [0.750–1.167]	0.553
					Codominant 1	0.895 [0.715–1.120]	0.334
					*Codominant 2*	*0.591 [0.375–0.933]*	*0.027*
(ii) *IL-12A*^**∗∗**^							
*rs2243115*	*T>G*						
	*TT*	*TG*	*GG*				
Control (*n* = 873)	746 (0.855)	120 (0.137)	7 (0.008)	0.077	*Additive*	*0.674 [0.499–0.909]*	*0.010*
					*Dominant*	*0.676 [0.494–0.925]*	*0.014*
					Recessive	0.294 [0.048–1.785]	0.183
pCAD (*n* = 1162)	1048 (0.902)	112 (0.096)	2 (0.002)	0.050	*Heterozygote*	*0.698 [0.508–0.956]*	*0.027*
					*Codominant 1*	*0.694 [0.505–0.954]*	*0.024*
					Codominant 2	0.282 [0.046–1.712]	0.169

^*∗*^Models were adjusted for age, gender, body mass index, current smoking status, alanine aminotransferase, aspartate aminotransferase, and uric acid. ^*∗∗*^Models were adjusted for age, gender, body mass index, and current smoking status. Italic numbers indicate significant associations. The control group subjects were normoglycaemic nondiabetic. MAF: minor allele frequency; pCAD: premature coronary artery disease.

Only the significant associated polymorphisms are shown.

**Table 4:** Association between *EBI3* and *IL-12A* gene polymorphisms and metabolic abnormalities in premature coronary artery disease patients.

Polymorphism	Genotype frequency *n* (%)			MAF	Model	OR [95% CI]	*P*
(i) *EBI3*							
*rs428253*	*G>C*						
	*GG*	*GC*	*CC*				
Alanine aminotransferase > p75					Additive	1.330 [1.083–1.632]	0.006
No (*n* = 590)	401 (0.679)	167 (0.283)	22 (0.038)	0.179	Dominant	1.429 [1.121–1.821]	0.004
Si (*n* = 572)	340 (0.594)	204 (0.357)	28 (0.049)	0.227	Heterozygote	1.392 [1.084–1.787]	0.010
					Codominant 1	1.425 [1.107–1.835]	0.006
Aspartate aminotransferase > p75							
No (*n* = 752)	489 (0.650)	238 (0.316)	25 (0.034)	0.191	Codominant 2	1.823 [1.022–3.250]	0.042
Si (*n* = 410)	251 (0.613)	135 (0.328)	24 (0.059)	0.223			
Type 2 diabetes mellitus					Dominant	0.753 [0.580–0.978]	0.033
No (*n* = 750)	458 (0.611)	259 (0.345)	33 (0.044)	0.217	Heterozygote	0.727 [0.554–0.954]	0.022
Si (*n* = 412)	282 (0.684)	112 (0.272)	18 (0.044)	0.180	Codominant 1	0.726 [0.522–0.955]	0.022
							
*rs4905*	*A>G*						
	*AA*	*AG*	*GG*				
Alanine aminotransferase > p75					Additive	1.241 [1.031–1.495]	0.023
No (*n* = 752)	400 (0.532)	302 (0.402)	50 (0.066)	0.267	Dominant	1.309 [1.037–1.653]	0.024
Si (*n* = 410)	212 (0.517)	162 (0.394)	36 (0.089)	0.285	Codominant 1	1.284 [1.006–1.640]	0.045
							
(ii) *IL-12A*							
*rs2243123*	*T>C*						
	*TT*	*TC*	*CC*				
Type 2 diabetes mellitus							
No (*n* = 750)	290 (0.387)	358 (0.477)	102 (0.137)	0.375	Recessive	1.148 [1.061–2.063]	0.021
Si (*n* = 412)	150 (0.365)	182 (0.441)	80 (0.194)	0.415	Codominant 2	1.511 [1.048–2.178]	0.028
							
*rs2243115*	*T>G*						
	*TT*	*TG*	*GG*				
Metabolic syndrome					Additive	0.591 [0.386–0.905]	0.015
No (*n* = 327)	286 (0.875)	40 (0.122)	1 (0.003)	0.064	Dominant	0.590 [0.381–0.912]	0.017
Si (*n* = 835)	762 (0.913)	72 (0.086)	1 (0.001)	0.044	Heterozygote	0.599 [0.386–0.929]	0.022
					Codominant 1	0.592 [0.385–0.927]	0.021
*rs583911*	*A>G*						
	*AA*	*AG*	*GG*				
Inflammation							
No (*n* = 930)	255 (0.274)	433 (0.466)	242 (0.260)	0.493	Recessive	0.633 [0.435–0.921]	0.017
Si (*n* = 232)	67 (0.287)	123 (0.532)	42 (0.181)	0.446			
Aspartate aminotransferase > p75					Additive	1.236 [1.046–1.460]	0.013
No (*n* = 712)	211 (0.296)	343 (0.482)	158 (0.222)	0.463	Dominant	1.318 [1.004–1.730]	0.046
Si (*n* = 450)	109 (0.242)	217 (0.483)	124 (0.275)	0.517	Recessive	1.344 [1.021–1.769]	0.035
					Codominant 2	1.529 [1.096–2.133]	0.013
GGT > p75							
No (*n* = 625)	178 (0.285)	309 (0.494)	138 (0.221)	0.468	Recessive	1.329 [1.011–1.748]	0.042
Si (*n* = 537)	141 (0.263)	251 (0.468)	145 (0.269)	0.504			

Table shows the models with significant associations. Models were adjusted for age, gender, and body mass index. MAF: minor allele frequency; GGT: gamma-glutamyl transferase.

**Table 5:** Association between *EBI3* and *IL-12A* gene polymorphisms and metabolic abnormalities in the control group.

Polymorphism	Genotype frequency *n* (%)			MAF	Model	OR [95% CI]	*P*
(i) *EBI3*							
*rs428253*	*G>C*						
	*GG*	*GC*	*CC*				
Hyperuricemia							
No (*n* = 726)	454 (0.625)	220 (0.303)	52 (0.072)	0.223	Heterozygote	1.595 [1.064–2.389]	0.024
Si (*n* = 147)	82 (0.555)	57 (0.390)	8 (0.055)	0.248	Codominant 1	1.567 [1.038–2.365]	0.032
							
*rs4740*	*G>A*						
	*GG*	*GA*	*AA*				
Central obesity							
No (*n* = 196)	99 (0.503)	78 (0.400)	19 (0.097)	0.296	Heterozygote	0.391 [0.163–0.937]	0.035
Si (*n* = 677)	377 (0.557)	251 (0.371)	49 (0.072)	0.258	Codominant 2	0.386 [0.157–0.949]	0.038
AST > p75					Additive	1.250 [1.004–1.557]	0.046
No (*n* = 567)	327 (0.576)	197 (0.348)	43 (0.076)	0.250	Dominant	1.430 [1.076–1.899]	0.014
Si (*n* = 306)	148 (0.485)	133 (0.433)	25 (0.082)	0.299	Heterozygote	1.433 [1.073–1.913]	0.015
					Codominant 1	1.473 [1.093–1.985]	0.011
*rs4905*	*A>G*						
	*AA*	*AG*	*GG*				
Central obesity							
No (*n* = 196)	99 (0.503)	78 (0.400)	19 (0.097)	0.296	Recessive	0.404 [0.170–0.960]	0.040
Si (*n* = 677)	374 (0.552)	253 (0.371)	50 (0.074)	0.261	Codominant 2	0.403 [0.165–0.983]	0.046
AST > p75					Dominant	1.399 [1.054–1.858]	0.020
No (*n* = 567)	324 (0.571)	199 (0.352)	44 (0.078)	0.253	Heterozygote	1.410 [1.056–1.882]	0.020
Si (*n* = 306)	148 (0.485)	133 (0.433)	25 (0.082)	0.299	Codominant 1	1.445 [1.072–1.946]	0.016
							
(ii) *IL-12A*							
*rs568408*	*G>A*						
	*GG*	*GA*	*AA*				
Metabolic syndrome							
No (*n* = 614)	542 (0.883)	69 (0.112)	3 (0.005)	0.061	Additive	0.583 [0.347–0.981]	0.042
Si (*n* = 259)	237 (0.915)	21 (0.081)	1 (0.004)	0.044			
							
*rs583911*	*A>G*						
	*AA*	*AG*	*GG*				
SAT > p75							
No (*n* = 483)	129 (0.268)	222 (0.459)	132 (0.273)	0.503	Heterozygote	1.776 [1.203–2.622]	0.004
Si (*n* = 390)	88 (0.226)	220 (0.563)	82 (0.211)	0.492	Codominant 1	1.776 [1.107–2.849]	0.017

Table shows the models with significant associations. Models were adjusted for age, gender, and body mass index. MAF: minor allele frequency, AST: aspartate aminotransferase, and SAT: subcutaneous adipose tissue.

**Table 6:** Interleukin 35 plasma concentrations in the study groups according to the *EBI3 *and *IL-12A* polymorphisms.

Polymorphism	Genotype	Controls (*n* = 447)		*P* ^*∗*^	pCAD (*n* = 447)		*P*
		*n*	Concentration (pg/mL)		*n*	Concentration (pg/mL)	
(i) *EBI3*							
rs428253	*GG*	275	2.72 [0.88–4.97]		286	3.16 [1.03–6.51]	
	*GC*	151	3.00 [1.62–5.23]	0.273	140	3.40 [1.19–7.76]	0.433
	*CC*	21	1.98 [0.19–5.10]		21	2.20 [1.63–3.78]	
rs4740	*GG*	*250*	*2.52 [0.88–4.50]*		233	3.23 [1.62–7.61]	
	*GA*	*160*	*3.00 [1.63–5.23]*	*0.020*	185	3.16 [0.95–6.15]	0.311
	*AA*	*37*	*3.40 [0.88–7.90]*		29	3.16 [1.62–5.23]	
rs4905	*AA*	*248*	*2.52 [0.88–4.40]*		233	3.23 [1.62–7.61]	
	*AG*	*164*	*3.00 [1.63–5.23]*	*0.017*	186	3.16 [0.95–6.15]	0.338
	*GG*	*35*	*3.78 [0.88–7.90]*		28	3.08 [1.62–5.23]	
(ii) *IL12A*							
rs2243115	*TT*	389	2.72 [0.88–4.97]		407	3.16 [1.62–6.44]	
	*TG + GG*	58	3.08 [0.83–5.23]	0.376	40	3.56 [0.38–7.95]	0.985
rs568408	*GG*	398	2.81 [0.88–4.97]		396	3.23 [1.62–6.74]	
	*GA + AA*	49	2.32 [0.31–7.17]	0.763	51	2.72 [0.95–6.44]	0.254
rs2243123	*TT*	188	2.72 [0.59–4.97]		183	3.40 [1.03–7.09]	
	*TC*	198	2.90 [0.88–5.23]	0.702	190	3.00 [1.62–6.31]	0.714
	*CC*	61	3.00 [0.88–5.23]		74	3.32 [1.47–8.38]	
rs583911	*AA*	113	3.00 [0.88–5.23]		130	3.23 [1.30–8.56]	
	*AG*	224	2.90 [0.88–5.23]	0.570	194	3.00 [0.95–6.19]	0.215
	*GG*	110	2.46 [0.88–4.82]		123	3.40 [1.62–7.09]	

Data are shown as median [interquartile range]. Comparisons were made using Mann–Whitney *U* test or Kruskal-Wallis test as appropriate. Italic numbers indicate significant associations.

pCAD: premature coronary artery disease.
